# Metabolic reprogramming in cancer: mechanistic insights from *Drosophila*

**DOI:** 10.1242/dmm.048934

**Published:** 2021-07-09

**Authors:** Kenneth Kin Lam Wong, Esther M. Verheyen

**Affiliations:** 1 Department of Molecular Biology and Biochemistry, Simon Fraser University, Burnaby, British Columbia, V5A 1S6, Canada; 2 Centre for Cell Biology, Development and Disease, Simon Fraser University, Burnaby, British Columbia, V5A 1S6, Canada

**Keywords:** Aerobic glycolysis, *Drosophila* cancer models, Metabolic reprogramming, Mitochondria

## Abstract

Cancer cells constantly reprogram their metabolism as the disease progresses. However, our understanding of the metabolic complexity of cancer remains incomplete. Extensive research in the fruit fly *Drosophila* has established numerous tumor models ranging from hyperplasia to neoplasia. These fly tumor models exhibit a broad range of metabolic profiles and varying nutrient sensitivity. Genetic studies show that fly tumors can use various alternative strategies, such as feedback circuits and nutrient-sensing machinery, to acquire and consolidate distinct metabolic profiles. These studies not only provide fresh insights into the causes and functional relevance of metabolic reprogramming but also identify metabolic vulnerabilities as potential targets for cancer therapy. Here, we review the conceptual advances in cancer metabolism derived from comparing and contrasting the metabolic profiles of fly tumor models, with a particular focus on the Warburg effect, mitochondrial metabolism, and the links between diet and cancer.

## Abstract (precis)

**Summary:** Recent research in fruit flies has demonstrated that tumors rewire their metabolism by using diverse strategies that involve feedback regulation, nutrient sensing, intercellular or even inter-organ interactions, yielding new molecules as potential cancer markers or drug targets.

## Introduction

Metabolic reprogramming is a key hallmark of cancer (Faubert et al., 2020). A frequently seen metabolic shift is the Warburg effect that describes the vigorous glucose uptake to fuel glycolysis and secretion of lactate by cancer cells, even in the presence of oxygen (Warburg et al., 1927). The discovery of the Warburg effect not only laid the basis for 2-deoxy-2-(^18^F)fluoro-D-glucose positron emission tomography (FDG-PET) in cancer diagnosis but also sparks continuing research in cancer metabolism, ranging from glycolysis and mitochondrial metabolism to nutrient-sensing machinery (Danhier et al., 2017). Notably, the recent emergence of nutrient-dependent post-translational modifications (PTMs), such as *O-*GlcNAcylation and lactylation, have advanced our concepts about the versatility of nutrients. In addition to being fuels and building blocks of macromolecules, metabolites can act as nutrient sensors to regulate signal transduction and transcription (Hart, 2019; Zhang et al., 2019). Targeting the metabolic vulnerabilities of cancer through either dietary or pharmacological interventions might thus represent promising approaches for cancer therapy (Kanarek et al., 2020).

**Fig. 1.**
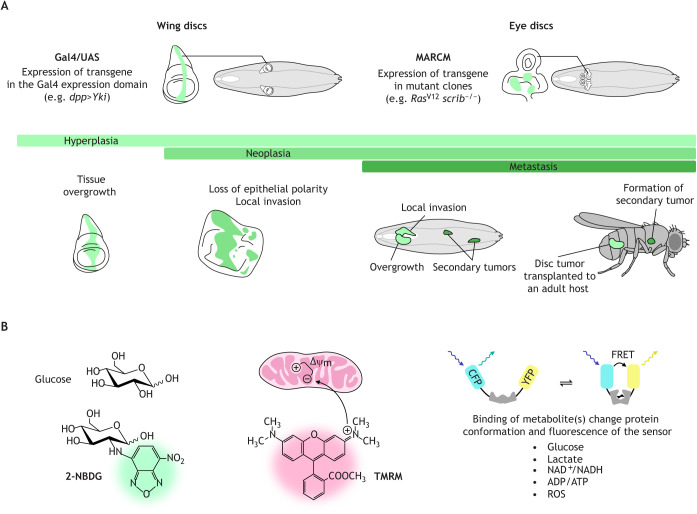
**Genetic tools and fluorescent probes to characterize the metabolic profiles of *Drosophila* tumor models.** (A) Imaginal disc epithelia to model human carcinomas. By using genetic techniques, such as the *Gal4/UAS* system and the MARCM technique, fly geneticists can easily and precisely manipulate the expression of oncogenes and/or tumor suppressor genes in certain cells of the larval imaginal wing and eye epithelial discs (green) to model human carcinomas. These cells can be marked with GFP or other fluorescent proteins for easy imaging. Numerous fly tumor models have been developed, which can be categorized into hyperplasia (overgrowth), neoplasia (overgrowth with local invasion and loss of epithelial cell polarity) and metastasis (neoplastic growth with secondary tumor formation). *dpp>yki* and *Ras^V12^ scrib^–/–^* are examples of hyperplasia and metastasis tumor models, respectively. (B) Fluorescent probes for imaging and characterization of metabolic profiles. Fluorescent dyes can be used directly to stain live or fixed tissues to characterize cell metabolism. For instance, the fluorescent glucose analog 2-NBDG is used to monitor glucose uptake. TMRM, which is a membrane-permeable fluorescent cation, accumulates in the hyperpolarized mitochondrial matrix that is more negative, thus enabling measurements of the mitochondrial membrane potential (ΔΨ_m_). To determine the intracellular levels of metabolites, biosensors can be used; the biosensors change their protein conformation and FRET-ratios upon binding to their specific metabolites. See Box 1 for more details. 2-NBDG, 2-[*N*-(7-nitrobenz-2-oxa-1,3-diazol-4-yl)amino]-2-deoxy-D-glucose; CFP, cyan fluorescent protein; Dpp, decapentaplegic; FRET, Förster resonance energy transfer; GFP, green fluorescent protein; MARCM, mosaic analysis with a repressible cell marker; Scrib, scribble; TMRM, tetramethylrhodamine, methyl ester; UAS, upstream activating sequence; YFP, yellow fluorescent protein; Yki, Yorkie.

The fruit fly *Drosophila* undergoes metamorphosis from the larval stage to adulthood (Markow, 2015). Although the fly is an invertebrate, it has proven to be a powerful genetic model organism for studying human cancer, largely owing to the strong conservation of genes and signaling cascades between humans and flies, and the reduced genetic redundancy in flies (Mirzoyan et al., 2019). As this Review focuses on *Drosophila* cancer mechanisms, we refer to fly nomenclature throughout. In *Drosophila* larvae, imaginal discs – such as wing and eye discs that give rise to wing and eye structures in adult flies, respectively – are monolayered epithelia with well-defined apical-basal polarity (Fig. 1A, top) (Beira and Paro, 2016). Thanks to robust genetic tools, there are multiple ways to achieve precise genetic manipulation within these structures. For instance, using the Gal4/UAS system (Brand and Perrimon, 1993), overexpression of the proto-oncogene Yorkie [Yki; Yes-associated protein (YAP) in vertebrates] in the *decapentaplegic* (*dpp*)-Gal4 expression domain results in a hyperplasia wing disc model (abbreviated genotype: *dpp>Yki*; hereafter referred to as *Yki* hyperplasia) (Huang et al., 2005; Wang et al., 2016). In another example, the technique mosaic analysis with a repressible cell marker (MARCM) (Lee and Luo, 1999) was used to induce expression of activated Ras in clones carrying homozygous mutations for the cell polarity gene *scribble* (*scrib*) in order to develop a neoplasia eye disc model (hereafter referred to as *Ras^V12^ scrib^−/−^* neoplasia) (Pagliarini and Xu, 2003). These tools allow us to study the cooperation of oncogenes and tumor suppressor genes, as well as their cell autonomous and non-autonomous effects on signaling and metabolic pathways *in vivo* (Mirzoyan et al., 2019). As malignancies of epithelial origin account for the majority of human cancers, larval imaginal discs provide an excellent platform to model epithelial tumors at multiple stages of their development (Herranz et al., 2016). Indeed, numerous fly tumor models have been established (Table 1). These include (1) hyperplasia, here defined as increased cell proliferation without loss of epithelial cell polarity; (2) neoplasia, defined as hyperplastic growth with disrupted epithelial cell polarity and local tumor invasion and; (3) metastasis, defined as neoplastic growth with secondary tumor formation (Fig. 1A, bottom).

**Table 1.**
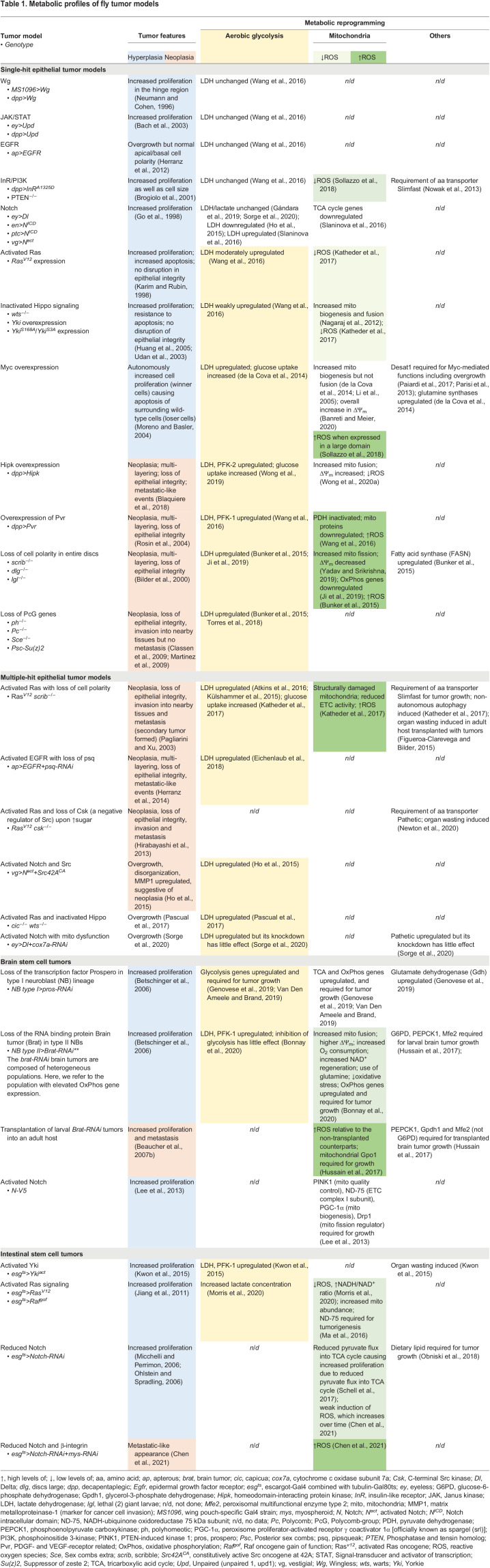
Metabolic profiles of fly tumor models

The past few years, especially 2015 to 2020, have seen tremendous progress in the field of fly tumor metabolism (some reviewed by Herranz and Cohen, 2017). We believe there are several reasons for the accelerating pace. First, most fly tumor models have been extensively characterized with respect to their aberrant cell signaling networks (Richardson and Portela, 2018; Sonoshita and Cagan, 2017). Second, diabetic fly models have been established (Graham and Pick, 2017), permitting investigation of links between diet and cancer. Third, as a whole-animal model, *Drosophila* permits the study of long-range, systemic metabolic changes. Fourth, newly developed metabolic probes enable detailed characterization of tumor metabolism *in vivo* (Fig. 1B; see Box 1, Using fluorescent probes to characterize metabolic profiles).

Three molecules – glucose, lactate and oxygen – are integral parts of the Warburg effect (DeBerardinis and Chandel, 2020). Here, we review the conceptual advances in cancer metabolism derived from studying the metabolic profiles of *Drosophila* tumor models (Table 1). There are three main themes in this Review: (1) the Warburg effect – how aerobic glycolysis is sustained in fly tumors and why; (2) the links between diet and cancer – how diet influences tumor progression and; (3) mitochondrial metabolism – whether and how mitochondrial metabolism is altered in tumors. This Review focuses on fly epithelial tumor models and touches on stem cell tumor models. As amino acid (aa), lipid and nucleotide metabolism in fly tumors remain understudied, these areas are not discussed. We also refer to the cited reviews for other aspects of cancer metabolism, such as organ wasting (cachexia) (Saavedra and Perrimon, 2019) and autophagy (Khezri and Rusten, 2019), as they are beyond the scope of this Review.

**Box 1. Using fluorescent probes to characterize metabolic profiles**

As metabolites and metabolic pathways are highly conserved between flies and mammals (Rajan and Perrimon, 2013), many established tools, such as fluorescent dyes and fluorescence resonance energy transfer (FRET)-based metabolite sensors to probe metabolic changes in cultured mammalian cells can be directly applied to *Drosophila* studies. A commonly used molecule to evaluate glucose uptake is the glucose analog 2-[N-(7-nitrobenz-2-oxa-1,3-diazol-4-yl)amino]-2-deoxy-D-glucose (2-NBDG), which possesses the fluorophore at carbon 2. Like D-glucose normally taken up by cells and fluorodeoxy-glucose (FDG) used in PET scans, 2-NBDG is taken up by cells and phosphorylated by hexokinases and, thus, becomes trapped within the cell (Yoshioka et al., 1996). Accumulation of 2-NBDG in the cell indicates a robust uptake of glucose. The combined use of 2-NBDG and FRET-based sensors to measure the relative intracellular levels of individual metabolites, such as glucose (Volkenhoff et al., 2018), pyruvate or lactate (Gándara et al., 2019), will be useful to estimate the glycolytic flux.

Specific probes have been developed for mitochondrial metabolism. Tetramethylrhodamine methyl ester (TMRM; or other variants like MitoTracker Red), which is a membrane-permeable fluorescent cation, accumulates in hyperpolarized mitochondrial matrix (which is more negative) but not in depolarized mitochondrial matrix, thus revealing the mitochondrial membrane potential (ΔΨ_m_) (Perry et al., 2011). However, there are several caveats to the use of TMRM for ΔΨ_m_ measurement. First, TMRM accumulation in mitochondria depends on both ΔΨ_m_ and mitochondrial mass, so normalization with mitochondrial mass is needed to assess ΔΨ_m_. Second, mitochondrial membrane hyperpolarization can be achieved by pumping protons into the intermembrane space either normally by ETC complexes I and III or abnormally by OxPhos complex V (ATP synthase). Genetic or pharmacological manipulations are needed to distinguish these two possible scenarios. Mitochondrial ETC complexes I and III are the main sites of ROS production (Murphy, 2009). A commonly used dye for measuring intracellular ROS is dihydroethidium (DHE), which fluoresces upon oxidation by superoxide and other non-specific species (Scaduto and Grotyohann, 1999). Given that ROS can be produced at other subcellular locations, including the endoplasmic reticulum (Leadsham et al., 2013), MitoSOX, a modified version of DHE that possesses a mitochondrion-targeting group, can be used to distinguish the sources of ROS (Dikalov and Harrison, 2014). Finally, FRET-based sensors for ROS, ATP/ADP (Tantama et al., 2013), NADH/NAD^+^ (Zhao et al., 2015), α-KG (Gándara et al., 2019) have also been used in fly studies. It is important to note that each probe has its strengths and pitfalls. To gain an accurate picture of the cell metabolism, use of single probes should be avoided, and other assays such as metabolic tracing and oxygen consumption assay may be considered.

## The Warburg effect

**Fig. 2.**
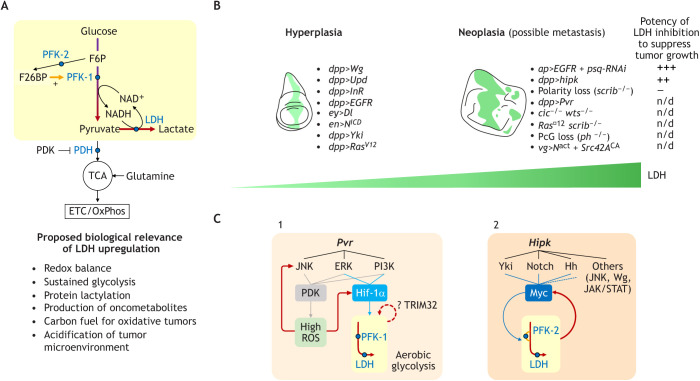
**Neoplastic tumor models manifest robust Warburg effect strengthened by signaling feedback.** (A) The Warburg effect (also known as aerobic glycolysis; top, shaded in light yellow). Metabolites are shown in black and metabolic enzymes in blue. F26BP acts as a potent allosteric activator of PFK-1 to stimulate glycolytic flux. Pyruvate, which is normally oxidized by PDH in mitochondria, is reduced to lactate by LDH when aerobic glycolysis takes place. A primary role of LDH is to regenerate NAD^+^ such that glycolysis can continue unabated. Additional roles of LDH have been proposed (bottom). (B) Robust LDH upregulation is a hallmark of neoplastic tumors. LDH upregulation is a hallmark of the Warburg effect. LDH levels in fly tumor models thus inform us to what extent the Warburg effect manifests. Although hyperplasia tumor models in general show no or only weak upregulation of LDH, neoplasia models display robust LDH upregulation. Fly tumor models for which LDH expression levels have been reported in the literature are listed in Table 1. The effect of LDH inhibition on suppressing tumor growth in different tumors varies. (C) Mechanisms that drive and sustain aerobic glycolysis. Mechanistic insights into how the Warburg effect arises and is reinforced in fly tumor models. In *Pvr* neoplasia (1), Hif-1α activated by ERK and PI3K induces upregulation of glycolytic genes, such as PFK-1 and LDH. Hif-1α is further activated by the JNK-ROS amplification loop. In *Hipk* neoplasia (2), Myc is upregulated via multiple perturbed pathways to promote glycolytic gene expression. Aerobic glycolysis and Myc constitute a positive feedback to consolidate the metabolic shift. ERK, extracellular signal-regulated kinase; ETC, electron transport chain; F26BP, fructose 2,6-bisphosphate; F6P, fructose 6-phosphate; Hh, Hedgehog; JAK/STAT, Janus kinase/signal transducer and activator of transcription; JNK, Jun N-terminal kinase; LDH, lactate dehydrogenase; n/d, not determined; NAD^+^/NADH, oxidized and reduced forms of nicotinamide adenine dinucleotide; OxPhos, oxidative phosphorylation; PDH, pyruvate dehydrogenase; PDK, PDH kinase; PFK-1, phosphofructokinase-1; PFK-2, phosphofructokinase-2/fructose 2,6-bisphosphatase; PI3K, phosphoinositide 3-kinase; Hif-1α, hypoxia-inducible factor 1 alpha; TCA, tricarboxylic acid; TRIM32, tripartite motif-containing protein 32; Wg, Wingless.

Glycolysis is a ten-step metabolic pathway oxidizing glucose to pyruvate (Fig. 2A, top). Normally, pyruvate is further oxidized in the tricarboxylic acid (TCA) cycle (also known as the citric acid cycle or Krebs cycle) within the mitochondrial matrix. In aerobic glycolysis, pyruvate is converted to lactate in the cytosol. This step is catalyzed by lactate dehydrogenase (LDH). As increased LDH is a general hallmark of aerobic glycolysis, we here use upregulation of LDH as an indicator of the Warburg effect to summarize its causes and consequences in fly tumor models. We also highlight the features of two key glycolytic enzymes – phosphofructokinase-1 (PFK-1) and phosphofructokinase-2/fructose 2,6-bisphosphatase (PFK-2/FBPase-2 or simply PFK-2, encoded by *PFKFB1-4* in vertebrates and *pfrx* in flies). Note that PFK-1 and PFK-2 are distinct enzymes, not isoforms, even though they share the same substrate fructose 6-phosphate (F6P).

### LDH

In *Drosophila*, LDH is encoded by a single gene called *Ldh* (also known as *ImpL3*), which is predominantly expressed in larval body-wall muscles and to some extent in the larval brain and salivary glands (Rechsteiner, 1970a; Wang et al., 2016). Notably, *Drosophila* LDH and one of its vertebrate orthologs LDHA share similar kinetic properties, preferring lactate production (Rechsteiner, 1970b). When pyruvate is reduced to form lactate, NADH – the reduced form of nicotinamide adenine dinucleotide (NAD) – is oxidized to NAD^+^, thus maintaining the redox balance and ensuring unabated glycolysis (Liberti and Locasale, 2016) (Fig. 2A, bottom).

#### LDH upregulation is more prevalent in neoplasia than in hyperplasia

Endogenous LDH is barely detectable in larval wing and eye disc epithelia (Rechsteiner, 1970a; Wang et al., 2016). Individual activation of oncogenic signaling pathways – including Wingless (Wg)/Wnt, Janus kinase/signal transducer and activator of transcription (JAK/STAT), insulin-like receptor/phosphoinositide 3-kinase (InR/PI3K) and epidermal growth factor receptor (EGFR) – in these epithelia leads to hyperplasia but not to LDH upregulation (Wang et al., 2016) (Fig. 2B, Table 1). Disc overgrowths caused by activated Yki and Ras exhibit weak and moderate LDH upregulation, respectively (Wang et al., 2016). Unchanged, increased and decreased levels of LDH/lactate have been reported in hyperplastic discs caused by activation of Notch (Gándara et al., 2019; Ho et al., 2015; Slaninova et al., 2016; Sorge et al., 2020). Such an inconsistency could be explained by the degree and extent of Notch activation, or might be a result of interactions with local signaling pathways. In addition to maintaining redox balance, the Warburg effect has been proposed to benefit cancer cells by promoting rapid synthesis of ATP, macromolecule biosynthesis, acidification of tumor microenvironment and by altering histone acetylation (Liberti and Locasale, 2016) (Fig. 2A, bottom). The observations that LDH expression is not usually induced in fly hyperplasia suggests that the metabolic demand for hyperplastic growth has not exceeded the capacity of glycolysis operating at physiological rate, or that the demand can be met by engaging alternative metabolic pathways.

In contrast to hyperplasia, robust LDH upregulation is evident in most, if not all, neoplastic disc tumor models (Atkins et al., 2016; Bunker et al., 2015; Eichenlaub et al., 2018; Hamaratoglu and Atkins, 2020; Ho et al., 2015; Külshammer et al., 2015; Pascual et al., 2017; Torres et al., 2018; Wang et al., 2016; Wong et al., 2019). Larvae bearing tumors usually experience a delay in the timing of pupation and the tumors continue to grow in the extended larval phase (Menut et al., 2007). Herranz et al. found that overexpression of EGFR with the simultaneous deletion of the chromatin regulator pipsqueak (psq), i.e. the *EGFR+psq^RNAi^* genotype, leads to massive tissue overgrowth in *Drosophila* (Herranz et al., 2012). Moreover, temporal analyses of these *EGFR+psq^RNAi^* tumors revealed a progressive increase in LDH expression (Eichenlaub et al., 2018). Collectively, these studies suggest that (1) LDH upregulation, indicative of the Warburg effect, is common in neoplasia but is not a feature of hyperplasia; (2) activation of a single oncogenic pathway is insufficient to cause robust LDH expression and; (3) tumor metabolism is dynamic, allowing the Warburg effect to be reinforced over time.

#### LDH drives neoplastic transformation in certain tumor types

To study the roles of LDH in tumor growth in *Drosophila*, researchers have used genetic manipulations to modulate *Ldh* gene expression. Overexpression of *Ldh* can transform *Egfr*-induced hyperplasia into neoplasia, as seen with other hyperplasia models, including those driven by Notch and Yki activation (Eichenlaub et al., 2018; Sorge et al., 2020). Genetic inhibition of LDH, however, has varying effects on tumor progression (Fig. 2B, right). In *EGFR psq-RNAi* neoplasia, knockdown of LDH reduces tumor size and prevents neoplastic transformation (Eichenlaub et al., 2018). However, in neoplasia due to overexpression of homeodomain-interacting protein kinase (*Hipk*) or *scrib^−/−^*, respectively, knockdown of LDH has only minor implications or no obvious effects (Bunker et al., 2015; Wong et al., 2019). In tumors induced by overexpression of PDGF- and VEGF-receptor-related (*Pvr*), the direct roles of LDH have not been examined. However, knockdown of *similar* [*sima* in flies; hypoxia-inducible factor-1 alpha, HIF1A (hereafter referred to as Hif-1α) in mammals], which strongly suppresses the upregulation of LDH along with other glycolytic genes, reduces tumor size (Wang et al., 2016). The different outcomes regarding LDH inhibition might result from a variability in knockdown efficiency in each tumor model or might reflect unique LDH requirements and/or a unique metabolic profile for tumor progression. Moreover, it would be interesting to see whether any compensatory mechanisms, e.g. glycerol 3-phosphate dehydrogenase – that also functions in NAD^+^ regeneration (Li et al., 2019) – fulfill the roles of LDH and permit the growth of LDH-depleted tumors.

How does the Warburg effect drive neoplastic transformation? Besides the above-mentioned proposed roles of the Warburg effect, recent research has provided new insights. First, histones (Zhang et al., 2019) and glycolytic enzymes (Gaffney et al., 2020) can be modified with a lactyl moiety at lysine residues. This PTM, coined lactylation (or lactoylation), implies that lactate acts as a signaling molecule bridging epigenetic regulation and metabolism. Yet, whether the donor substrate for histone lactylation *in vivo* is lactyl-CoA derived from lactate or S-lactoylglutathione from methylglyoxal remains controversial (Kulkarni and Brookes, 2020 preprint). Second, LDH promotes the accumulation of the oncometabolite L-2-hydroxyglutarate (L-2HG) by directly catalyzing its synthesis from α-ketoglutarate (also known as 2-oxoglutarate, hereafter referred to as α-KG) and/or by indirectly using lactate to inhibit L-2HG dehydrogenase and hence L-2HG degradation (Intlekofer et al., 2015; Li et al., 2017). As an α-KG antagonist, L-2HG inhibits α-KG-dependent DNA and histone demethylases (Xu et al., 2011), and is associated with DNA and histone hypermethylation in renal cell carcinomas (Shelar et al., 2018; Shim et al., 2014). These studies did significantly broaden our understanding of the tumorigenic roles of LDH/lactate (summarized in Fig. 2A, bottom). Nonetheless, whether the Warburg effect contributes to neoplastic transformation in fly hyperplasia tumor models through inducing epigenetic changes remains an open question.

#### Signal divergence, convergence and feedback cause robust LDH upregulation

LDH expression in flies can be induced by hypoxia (Lavista-Llanos et al., 2002), mitochondrial dysfunction (Sorge et al., 2020), oncogenic drivers (Wang et al., 2016; Wong et al., 2019), growth hormones (Abu-Shumays and Fristrom, 1997; Li et al., 2018) and bacterial infection (Krejčová et al., 2019). Recent studies have used genetic analyses to dissect how aerobic glycolysis arises in *Pvr* and *Hipk* neoplasia models. In *Pvr* tumors, elevated levels of Pvr promote translation of Hif-1α through co-activation of extracellular signal-regulated kinase (ERK, encoded by the sole *Drosophila* ERK, *rolled*) and PI3K pathways (Wang et al., 2016) (Fig. 2C.1). This double-pathway activation, concurrent with Hif-1α buildup, is also observed in neoplasia induced by losses of *lethal (2) giant larvae* [*l(2)gl*, hereafter referred to as *lgl*] and ribosomal protein L27A (RpL27A) (Grifoni et al., 2015), suggesting that the pathway activation in *Pvr* tumors may be shared among tumors of different genetic makeups. A distinct mechanism is found in *Hipk* tumors (Wong et al., 2019) (Fig. 2C.2). Elevated Hipk, which is known to perturb multiple signaling pathways, including Hippo (Chen and Verheyen, 2012; Poon et al., 2012), Notch (Lee et al., 2009a), Wnt (Lee et al., 2009b), Hedgehog (Swarup and Verheyen, 2011) and JAK/STAT (Tettweiler et al., 2019) (reviewed by Blaquiere and Verheyen, 2017), promotes *Myc* transcription – probably through convergence of signals that control *Myc* expression. In these diverse contexts, accumulated Hif-1α or Myc activate the transcription of glycolytic genes, consistent with their conserved roles as glycolytic inducers in human cancers (Koppenol et al., 2011).

More importantly, recent work has uncovered how aerobic glycolysis can be sustained in tumor development. In *Pvr* tumors, elevated *Pvr* activates Jun N-terminal kinase (JNK, encoded by the sole *Drosophila* JNK, *basket*) signaling, followed by overproduction of reactive oxygen species (ROS) (Wang et al., 2016) (Fig. 2C.1). ROS feeds back to sustain JNK and Hif-1α activation, thus strengthening glycolysis. In *Hipk* tumors where ROS remain at a basal level (Wong et al., 2020a), Myc-induced aerobic glycolysis perpetuates Myc accumulation, forming a positive feedback loop (Wong et al., 2019) (Fig. 2C.2). A glycolysis-dependent amplification loop may exist in *Pvr* tumors too, as tripartite motif-containing protein 32 (TRIM32; encoded by *thin*), an E3 ubiquitin ligase that maintains glycolytic flux, is required for LDH transcription in tumors (Bawa et al., 2020). Nonetheless, how TRIM32 controls *LDH* at the transcriptional level in Pvr tumors warrants further investigation.

Taken together, these studies illustrate that aerobic glycolysis is not only driven by cooperation of more than one oncogenic pathway but also that it is reinforced by reciprocal stimulation of oncogenic and metabolic signals. The establishment of feedback circuits thus provides a plausible explanation for the incremental levels of LDH expression during tumor progression.

### PFK-1 and PFK-2

PFK-1 governs the committed, second irreversible step of glycolysis, catalyzing the phosphorylation of F6P to fructose 1,6-bisphosphate (Fig. 2A, top). PFK-2 is a bifunctional enzyme that catalyzes the reversible conversion of F6P to fructose 2,6-bisphosphate (F26BP). F26BP is a potent allosteric activator of PFK-1, thus boosting glycolysis. Vertebrate PFK-1 and its fly homolog have identical aa residues that can bind F26BP (Nunes et al., 2016), suggesting that the allosteric regulation of PFK-1 by F26BP is conserved. Through metabolite assays, several studies have confirmed that both PFK-1 and PFK-2 regulate glycolytic flux in flies (Havula et al., 2013; Li et al., 2018; Wong et al., 2019).

#### PFK-1 and PFK-2 activate oncogenic drivers, either directly or indirectly

Together with LDH, PFK-1 or PFK-2 are commonly upregulated in neoplasia tumor models, including *Pvr* (Wang et al., 2016), *Hipk* (Wong et al., 2019) and *Ras^V12^ scrib^−/−^* neoplasia (Wei et al., 2020 preprint). Recent studies in mammals revealed that PFK-1 and PFK-2 could play non-metabolic roles. For instance, in the nucleus PFK-1 binds to TEA domain transcription factors (TEADs), i.e. scalloped (*sd*) in flies, to stimulate the pro-tumorigenic functions of YAP (Yki in flies) (Enzo et al., 2015). Moreover, the PFK-2 isoform PFKFB4 directly phosphorylates and activates steroid receptor co-activator-3 (SRC-3), and the PFKFB4–SRC-3 axis drives primary growth and metastasis of basal-subtype breast cancer cells in a mouse xenograft model (Dasgupta et al., 2018). This indicates that PFK-2 is not only able to phosphorylate the metabolite F6P but also protein substrates.

Strikingly, regulation of transcription factors or co-factors by PFK-1 and PFK-2 is also observed in fly tumors. In *Yki lgl^−/−^* tumors, PFK-1 is required for the upregulation of Yki target genes, including *Myc* (Enzo et al., 2015). In *Hipk* tumors, both PFK-1 and PFK-2 are required to sustain Myc protein accumulation but neither is necessary for *Myc* transcription (Wong et al., 2019). Loss of either PFK-1 or PFK-2 suppresses the growth of both tumor models (Enzo et al., 2015; Wong et al., 2019). Although the exact molecular mechanisms in fly tumors need further investigation, these studies imply that PFK-1, PFK-2 or the associated glycolytic flux can modulate Myc at both transcriptional and post-transcriptional levels. Myc is a key cancer driver and, given that it is generally considered ‘undruggable’ due to its nuclear localization, one might want to exploit the metabolic control of Myc to slow down the progression of cancers, especially for those comprising amplification of Myc.

Taken together, recent fly studies have demonstrated that the Warburg effect plays a positive role in neoplastic transformation and can be reinforced over time by feedback circuits in epithelial tumor models. Although the Warburg effect has also been observed in fly stem cell tumor models (Table 1), our current understanding of its relevance in stem cell tumors is limited. We discuss more about the stem cell tumor models in the mitochondrial metabolism section (section 3) below.

## Links between diet and cancer

Having discussed the intrinsic induction of aerobic glycolysis in fly tumors, we next discuss a series of recent studies exploring how tumor progression is accelerated by an extrinsic factor – dietary sugar.

**Fig. 3.**
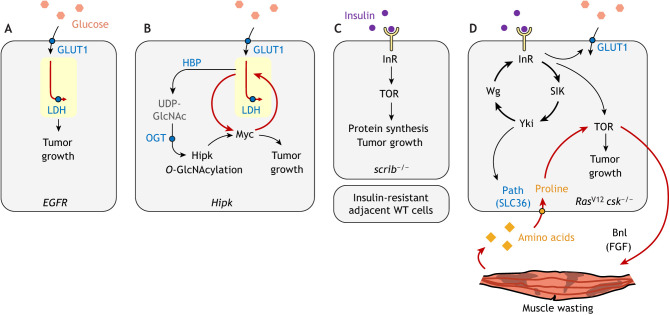
**Mechanistic links between diet and fly tumor progression.** Fly tumor cells take advantages of the metabolic pathways, nutrient-sensing machinery and systemic regulation to progress in response to elevated circulating levels of glucose as well as insulin upon dietary changes. (A) Upon a high-sugar diet, elevated glucose levels promote the transformation of EGFR hyperplasia into neoplasia in a LDH-dependent manner. (B) Upon a high-sugar diet, elevated glucose levels promote Hipk tumorigenesis through the HBP/OGT axis. Activated Hipk promotes upregulation of Myc and subsequent glucose uptake, which further stimulates the HBP flux. (C) Upon a high-protein diet, elevated insulin levels enable *scrib^−/−^* clones to proliferate instead of being eliminated. Mechanistically, insulin activates InR/TOR signaling that drives protein synthesis and confers growth advantages over the neighboring wild-type cells. (D) Upon a high-sugar diet, elevated insulin levels activate two tightly linked signaling loops in the *Ras^V12^ csk^−/−^* tumor model. This feedback circuitry drives metastasis-like behaviors cell autonomously and causes wasting phenotypes in distant organs, such as skeletal muscle. Bnl, branchless; FGF, fibroblast growth factor; GLUT1, glucose transporter 1; HBP, hexosamine biosynthetic pathway; InR, insulin receptor; OGT, *O-*GlcNAc transferase; Path, pathetic (SLC36 in mammals); SIK, salt-inducible kinase; TOR, target of rapamycin.

Considerable evidence from epidemiological studies show that diabetes and obesity pose an increased risk for cancer (reviewed by Gallagher and LeRoith, 2015). To study the connections between diet and cancer, one can take advantage of the well-defined diabetic model in *Drosophila*, established by feeding flies a high-sugar diet. This results in flies developing hyperglycemia, hyperinsulinemia, lipid accumulation in the fat body – which mimics obesity – and insulin resistance, thus recapitulating human type II diabetes (Musselman et al., 2011). By using this diabetic model, numerous studies have demonstrated that tumors exploit the versatile intracellular nutrient-sensing machinery to boost growth upon high-sugar feeding (Fig. 3). The expanding list of the mechanisms uncovered may help explain why diabetes appears to be a general risk factor for cancers despite their diverse genetic makeups.

### Elevated sugar promotes EGFR neoplastic transformation through LDH

As mentioned earlier, EGFR activation is only sufficient to cause hyperplasia (Herranz et al., 2012). When more glucose is available to *Egfr*-overexpressing cells by either overexpressing the glucose transporter GLUT1 or by feeding the larvae a high-sugar diet, the hyperplasia advances to neoplasia (Fig. 3A) (Eichenlaub et al., 2018). This glucose-mediated effect depends on LDH activity, suggesting that the high-sugar diet drives neoplastic transformation through elevated aerobic glycolysis (Eichenlaub et al., 2018). How does high sugar activate glycolysis? One key intracellular sugar sensor is the highly conserved transcription factor carbohydrate response element binding protein (MLXIPL, also known as and hereafter referred to as ChREBP in mammals; Mondo in flies) (Chng et al., 2017). Once activated in response to high levels of sugar, it binds with its partner, the transcription regulator Mlx, in the nucleus and activates the expression of glycolytic genes, such as *PFK-2* and sugar transporters but not *LDH* (Havula et al., 2013; Mattila et al., 2015). To date, little is known about the contributions of ChREBP/Mlx to sugar induced-tumor growth in the EGFR hyperplasia model.

### Elevated sugar potentiates Hipk tumorigenesis through the HBP/OGT axis

The hexosamine biosynthetic pathway (HBP) is a branch of glycolysis that diverts F6P to yield the end-product of HBP, uridine diphosphate-*N*-acetylglucosamine (UDP-GlcNAc) (Akella et al., 2019). By using this nucleotide-activated monosaccharide, *O-*GlcNAc transferase (OGT) catalyzes the addition *O-*GlcNAc to serine and threonine residues of intracellular proteins – a PTM termed *O-*GlcNAcylation. The catalytic activity of OGT depends on the intracellular concentration of UDP-GlcNAc (Kreppel and Hart, 1999) that, in turn, depends on the abundance of glucose as well as of other metabolites required in the HBP, i.e. glucosamine, acetyl-CoA and UTP (Yang and Qian, 2017). Thus, *O*-GlcNAc is regarded as a molecular switch for nutrient sensing, allowing the cell to sense and respond to nutrient availability (Hart, 2019).

A growing number of cancer-related proteins are *O-*GlcNAc modified, for instance, MYC (Chou et al., 1995a,b) and the core components of Hippo signaling, including YAP (Peng et al., 2017; Zhang et al., 2017) and LATS2 (Kim et al., 2020). OGT also *O-*GlcNAcylates the fly proto-oncogene Hipk and human HIPK2 and promotes their stability (Wong et al., 2020b). Feeding *Hipk*-overexpressing larvae a high-sucrose diet potentiates the tumorigenic activities of Hipk through the HBP/OGT axis (Fig. 3B). Intriguingly, on a normal diet, Hipk overexpression induces mild upregulation of OGT and a Myc-driven increase in glucose uptake (Wong et al., 2020b, 2019). This suggests that a positive feedback loop – OGT-Hipk-OGT – exists to maximize the nutrient sensing capacity and sustain Hipk buildup, similar to the feedback regulation between OGT and YAP reported in human pancreatic and liver cancer cells (Peng et al., 2017; Zhang et al., 2017). To the best of our knowledge, *O*-GlcNAc profiling has only been used to study development of wild-type *Drosophila* (Selvan et al., 2017). Whether *O-*GlcNAcylation has broader roles in tumorigenesis remains a fertile ground for future studies. Analysis of the *O-*GlcNAc proteome in fly tumor models may provide fresh insights into the molecular links between nutrient sensing and cancer phenotypes.

### Elevated insulin promotes scrib^−/−^ tumorigenesis through InR activation

Loss of cell polarity genes, such as *scrib*, from the entire imaginal disc gives rise to neoplasia (Bilder et al., 2000). However, when a *scrib* mutation is introduced in random clones that are surrounded by wild-type cells, *scrib^−/−^* clones are eliminated from the epithelium (Brumby and Richardson, 2003). This phenomenon is known as cell competition. ‘Winner’ cells proliferate whereas ‘loser’ cells die. Cell competition models in flies and in mice allow us to glimpse at the early stage of carcinogenesis and study the molecular events that govern cell fitness surveillance (Bowling et al., 2019). Intriguingly, upon high-protein feeding, *scrib^−/−^* clones evade elimination (Sanaki et al., 2020) (Fig. 3C). Mechanistically, high-protein feeding, similar to high-sugar feeding, causes hyperinsulinemia, which activates expression of the insulin receptor (*InR*) and insulin signaling in *scrib^−/−^* clones. As a result, protein synthesis increases, and tumorigenesis arises (Sanaki et al., 2020). This study highlights that, in response to high insulin levels, *scrib^−/−^* clones retain insulin sensitivity in the insulin-resistant body, which is likely to be through upregulation of *InR*. Similar observations have been made in *Ras^V12^ C-terminal Src kinase (csk)^−/−^* tumors.

### Elevated sugar levels potentiate Ras*^V12^* csk^−/−^ metastasis through integrated signaling loops

Similar to *scrib^−/−^* clones, *Ras^V12^ csk^−/−^* clones surrounded by wild-type cells are always eliminated from the eye disc epithelia. Upon high-sugar feeding, these *Ras^V12^ csk^−/−^* clones not only evade cell competition and display neoplastic growth but also form secondary tumors, indicative of metastasis-like events (Hirabayashi et al., 2013) (Fig. 3D). The molecular underpinnings of the effect sugar has on *Ras^V12^ csk^−/−^* tumor progression have been elucidated in several studies (Hirabayashi et al., 2013; Hirabayashi and Cagan, 2015; Newton et al., 2020). Briefly, the *Ras^V12^ csk^−/−^* genotype synergizes with high-sugar feeding to activate salt-inducible kinases (SIKs) that function downstream of InR signaling (Choi et al., 2015). SIKs inhibit Hippo signaling, thus activating Yki (Wehr et al., 2013). Activated Yki increases expression of the Wg ligand that acts locally to stimulate Wg signaling to drive expression of *InR*. Increased InR expression, in conjunction with the diet-induced hyperinsulinemia, activates InR signaling. This forms an InR-SIK-Yki-Wg/InR signaling loop that potentiates glucose uptake, insulin sensitivity and secondary tumor formation. Moreover, via the canonical intracellular aa sensor InR-target of rapamycin (TOR) axis, this loop is linked to another positive feedback involving production and secretion of the fibroblast growth factor ligand Branchless (Bnl) to sustain muscle wasting (Newton et al., 2020). Muscle wasting supplies the tumors with proline that, in turn, strengthens TOR activation and drives tumor growth. Thus, in addition to confirming the well-known mechanistic roles of insulin in TOR activation and protein synthesis (Yang et al., 2017), these fly studies identified essential intracellular and inter-organ signaling networks that mediate insulin-induced tumor progression. Of note, the circulating growth factors or metabolites secreted under the action of insulin may serve as useful markers for cancer detection.

In *Drosophila*, *Ras^V12^ scrib^−/−^* is the most studied neoplasia model, which differs from the *Ras^V12^ csk^−/−^* model. First, unlike *Ras^V12^ csk^−/−^* or *scrib^−/−^* clones, *Ras^V12^ scrib^−/−^* clones do not undergo cell elimination (Brumby and Richardson, 2003). Instead, *Ras^V12^* expression helps *scrib^−/−^* clones evade cell competition. Second, it appears that high levels of sugar do not have any noticeable effect on *Ras^V12^ scrib^−/−^* tumor progression (Hirabayashi et al., 2013). These observations suggest that the effects of sugar are either tumor specific or that *Ras^V12^ scrib^−/−^* tumors have already acquired alternative metabolic processes, such that sugar is no longer a limiting factor for their progression.

Collectively, these fly studies demonstrate tumors can utilize LDH-dependent glycolysis, HBP-OGT or InR-TOR axes, or even inter-organ communications to progress during hyperglycemia or hyperinsulinemia.

## Mitochondrial metabolism

**Fig. 4.**
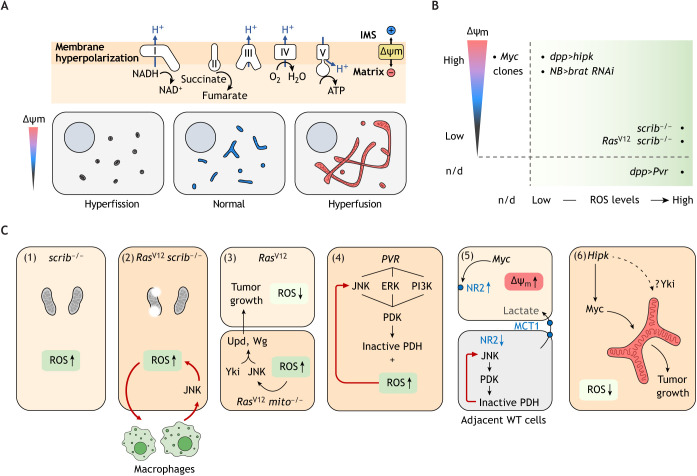
**Fly models of tumorigenesis display distinct mitochondrial profiles.** (A) Mitochondrial energetics and dynamics. Top: Schematic of the electron transport chain (ETC) in the inner mitochondrial membrane, showing ETC complexes I–V. When mitochondrial respiration is active, proton pumping into the IMS establishes a negative, i.e. hyperpolarized, membrane potential (ΔΨ_m_). Bottom: Mitochondria constantly undergo fission and fusion, normally in response to the metabolic needs of the cell and reflect the metabolic states. (B) Mitochondrial profiles of fly models based on ΔΨ_m_ and different levels of ROS. Only six models, i.e. *Myc*, *dpp>hipk*, *NB>brat-RNAi*, *scrib^−/−^*, *Ras^V12^ scrib^−/−^* and *dpp>Pvr*, are shown here because the mitochondrial profiles of others have not yet been described. Note that fly models comprising a high membrane potential (ΔΨ_m_) are always associated with low levels of ROS and those comprising a low ΔΨ_m_ with high levels of ROS. Notice that *NB>brat-RNAi* refers to the tumor population featuring high OxPhos and low levels of oxidative stress. (C) Distinct mitochondrial profiles in fly tumor models (1) *scrib^−/−^* tumors feature mitochondrial fission and high levels of ROS (ROS↑). (2) *Ras^V12^ scrib^−/−^* tumors accumulate ‘burst’ mitochondria and display high levels of ROS. The latter facilitate the recruitment of macrophages, which constitute an intercellular feedback circuit to drive tumor growth. (3) Mitochondrial dysfunction in *Ras^V12^ mito^−/−^* clones causes high levels of ROS, which results in cell senescence autonomously but increases the invasiveness of the neighboring *Ras^V12^* clones. (4) *Pvr* tumors feature inactivated mitochondrial pyruvate oxidation and high levels of ROS. The latter feeds back to JNK to strengthen the metabolic shift. (5) *Myc* clones show an overall increase in ΔΨ_m_ (ΔΨ_m_↑). Owing to a difference in NR2 levels in Myc clones and the neighboring wild-type (WT) clones, mitochondrial pyruvate oxidization is inactivated in WT clones, which then produce and transfer lactate to the Myc clones. (6) *Hipk* tumors feature low levels of ROS (ROS↓), mitochondrial fusion and hyperpolarization. Hyperpolarized mitochondria, regardless of the shape, drives tumor growth. IMS, intermembrane space; NR2, NMDA receptor subunit 2; Upd, Unpaired.

Mitochondria are the bioenergetic, biosynthetic and signaling hubs that host the TCA cycle, the electron transport chain (ETC) and the oxidative phosphorylation (OxPhos) pathway, the production of ROS, as well as numerous metabolic pathways involving fatty acids, aa and nucleotides (Fig. 4A) (Vyas et al., 2016). As highly dynamic organelles, mitochondria constantly undergo fission and fusion, mediated by three main regulators – the fission regulator Dynamin related protein 1 (Drp1; DNM1l in mammals), the inner membrane fusion regulator Optic atrophy 1 (Opa1) and the outer membrane fusion regulator Mitochondrial assembly regulatory factor (Marf; (mitofusin 1/2 Mfn1/2 in mammals) (Westermann, 2012). Often, fragmented mitochondria are damaged and susceptible to removal by autophagy. Fused mitochondria, by contrast, provide maximal respiratory capacity to meet surging metabolic demands. Thus, mitochondrial dynamics reflect the bioenergetic state of the cell, with active mitochondria comprising a difference of membrane potentials (ΔΨ_m_) across their membrane, i.e. a ΔΨ_m_ that is highly polarized (Westermann, 2012).

Warburg hypothesized that mitochondrial dysfunction is the cause of cancer (Warburg, 1956). In support of his hypothesis, several TCA enzymes, such as succinate dehydrogenase and fumarase were identified as tumor suppressors (Gottlieb and Tomlinson, 2005; King et al., 2006). However, accumulating evidence demonstrates that some cancer cells maintain and rely on TCA/OxPhos (Scott et al., 2011; Zu and Guppy, 2004). Thus, unlike aerobic glycolysis, which is universal in most cancer cells, mitochondrial metabolism in cancer is more heterogeneous and elusive than previously envisioned. Currently, the research on cancer mitochondria seems to lag behind that on the Warburg effect. One notable example is that PET imaging of OxPhos-dependent tumors has just recently been tested in mice (Momcilovic et al., 2019), whereas FDG-PET is widely applied in clinics.

### Fly tumor models with high levels of ROS or low membrane potential

Fly tumors exhibit a dynamic range of mitochondrial properties (Fig. 4B). Here, we first describe tumor models that feature excessive levels of ROS – presumably produced mainly by mitochondria, as well as membrane depolarization (Fig. 4C).

#### *scrib^−/−^* neoplasia

Wing disc tumors that entirely lack the cell polarity determinants *scrib*, *discs large* (*dlg*) or *lgl*, produce robust ROS levels (Bunker et al., 2015; Yadav and Srikrishna, 2019) (Fig. 4C.1). Scavenging ROS by overexpressing anti-oxidant enzymes has negligible effects on *dlg-RNAi* neoplasia, suggesting that ROS are dispensable for its growth (Bunker et al., 2015). High levels of ROS production can be caused by aberrant mitochondrial metabolism, for instance, by defects in ATP synthesis and a high NADH to NAD^+^ ratio (Murphy, 2009); it is, therefore, plausible that the tumor mitochondria are aberrant. Indeed, *scrib*-*RNAi* neoplasia features *Drp1* upregulation, *Marf* downregulation, mitochondrial hyperfission and membrane depolarization (Yadav and Srikrishna, 2019). Altered mitochondrial morphologies have been implicated in diverse aspects of cancer development in mammals, including tumor metabolism, cell death, proliferation, differentiation and migration (Kashatus, 2018). Nonetheless, whether changes in *Drp1* or *Marf* expression play a role in the mitochondrial hyperfission or the neoplastic growth of *scrib-RNAi* discs has not been investigated yet. Additionally, the roles of mitochondrial morphology seem to be tumor specific; mitochondrial fusion is required for growth of *brat-RNAi* brain tumors but not of *Hipk* epithelial tumors (for further details see sections ‘Hipk neoplasia’ and ‘Neural stem tumors’).

Temporal single-cell RNA-seq analyses reveal that, as *scrib^−/−^* tumors progress, both OxPhos and JNK are downregulated (Ji et al., 2019). In the early stage, high JNK activity inhibits the growth of *scrib^−/−^* tumor (Ji et al., 2019). Intriguingly, and probably counterintuitively, ETC genes, such as *cox5A*/*CoVa* (encoding an ETC complex IV subunit) and *ATPsyn-α*/*blw* (encoding an OxPhos complex V subunit) are required for JNK activation in *scrib*-depleted cells (Poernbacher and Vincent, 2018). Thus, it would be interesting to test whether restoring proper mitochondrial function helps prevent *scrib^−/−^* tumor initiation.

#### *Ras^V12^ scrib^−/−^* neoplasia

*Ras^V12^ scrib^−/−^* tumors accumulate structurally damaged mitochondria with breached (‘burst’) membranes together with normal-looking mitochondria (Katheder et al., 2017) (Fig. 4C.2). The reserve respiratory capacity of the tumor mitochondria is reduced, and high levels of ROS are produced in ways that depend on JNK and JAK/STAT (Katheder et al., 2017).

ROS in *Ras^V12^ scrib^−/−^* neoplasia can be produced in two different ways, extracellularly by the plasma membrane-bound NADPH oxidase (Duox) and intracellularly by mitochondria (Pérez et al., 2017). Functionally, ROS recruit and activate plasmatocytes, i.e. the fly macrophages (Fogarty et al., 2016), which release the Eiger ligand to activate JNK signaling in the tumors, thus constituting a positive feedback loop to promote tumor growth (Pérez et al., 2017).

In a similar *Drosophila* tumor model induced by *Ras^V12^ lgl^−/−^*, inhibition of mitochondrial ETC produces pleiotropic effects. Knockdown of *ND-51*, the fly ortholog of human *NDUFV1* (encoding an ETC complex I subunit) in the tumors reduces proliferation but stimulates macrophage recruitment (Kurelac et al., 2019). This is consistent with recent studies in mouse xenograft models demonstrating that ETC complexes I, II and III are all necessary for tumor growth by regenerating NAD^+^, FAD and ubiquinol, respectively (Martínez-Reyes et al., 2020), and that drugs targeting ETC  – such as metformin that blocks its complex I activity – may facilitate the recruitment of immune cells (Puschel et al., 2020). Thus, tumor mitochondria seem to be a double-edged sword. It is tempting to speculate that distinct populations of mitochondria – normal-looking and ‘burst’ mitochondria – in *Ras^V12^ scrib^−/−^* tumors work together to keep a balance between promoting primary tumor growth and creating a favorable microenvironment for metastatic spread. Also, these studies highlight the need of combinatorial therapy to simultaneously block ETC and the consequent inflammatory response (Kurelac et al., 2019).

#### Mitochondrial impairment in *Ras^V12^* cells induces non-autonomous effects on neighboring *Ras^V12^* cells

*Ras^V12^*, when expressed alone, causes benign hyperplastic growth. Although the mitochondrial profile of *Ras^V12^* hyperplasia has not been fully characterized, several studies have explored the interactions between *Ras^V12^* and mitochondrial dysfunction using the *Ras^V12^ mito^−/−^ // Ras^V12^* tumor model (Nakamura et al., 2014; Ohsawa et al., 2012) (Fig. 4C.3). This model is established by deliberately introducing mutations to disrupt mitochondrial functions in a subset of *Ras^V12^*-expressing cells. *mito^−/−^* denotes mitochondrial dysfunction caused by either *Pdsw^−/−^* (*Pdsw* encodes an ETC complex I subunit) or *CoVa^−/−^*. This model, therefore, mimics the stepwise accumulation of mutations and the rise of tumor heterogeneity as cancer evolves.

Intriguingly, *mito^−/−^* provokes cell senescence in *Ras^V12^ mito^−/−^* clones, so that their proliferation rate slows down (Nakamura et al., 2014). This indicates the requirement of proper mitochondrial function for *Ras^V12^* hyperplasia. More importantly, mitochondrial dysfunction in *Ras^V12^ mito^−/−^* clones triggers the senescence-associated secretory phenotype that transforms the neighboring *Ras^V12^* hyperplasia into aggressive neoplasia (Ohsawa et al., 2012). Mechanistically, *Ras^V12^* and *mito^−/−^* synergize to produce high ROS levels, which activates JNK signaling (Ohsawa et al., 2012). The cooperation of JNK and Yki signaling drives the production and secretion of Wg and the inflammatory cytokine Unpaired (Upd in flies; IL6 in vertebrates), which act on the nearby *Ras^V12^* hyperplasia to promote tumor growth (Ohsawa et al., 2012). Hence, this model further shows the opposing effects of perturbed mitochondrial metabolism – suppressing proliferation autonomously, while promoting non-autonomous neoplastic transformation.

#### *Pvr* tumors

*Pvr* tumors are another tumor type with elevated ROS (Wang et al., 2016) (Fig. 4C.4). In these tumors, pyruvate dehydrogenase (PDH) kinase (PDK) is activated by ERK, PI3K and JNK. PDK is a negative regulator of PDH (Fig. 2A, top). PDH catalyzes the irreversible oxidative decarboxylation of pyruvate to yield acetyl-CoA in the mitochondrial matrix. Therefore, PDH inactivation presumably attenuates the pyruvate flux into the TCA cycle and causes mitochondrial dysfunction. Consistent with this notion, these tumors produce excessive ROS in a PDK-dependent manner. The JNK/PDK/ROS/JNK feedback loop, as discussed above, reinforces this metabolic state. Such loop does not exist in *Ras^V12^ mito^−/−^* clones, which establish a *mito^−/−^*/ROS/JNK signaling axis instead. This suggests that inhibiting JNK signaling is no longer an option to suppress high ROS once mitochondria are irreversibly damaged. Nonetheless, whether *Pvr* tumor mitochondria are active or not remains inconclusive because cancer has bypass systems, i.e. by using glutamine or acetate in *brat-RNAi* brain tumors (see below), to keep mitochondrial flux operational (Bonnay et al., 2020).

### Fly tumor models with low levels of ROS or high membrane potential

In this section, we focus on those models that have a distinct mitochondrial profile – low levels of ROS or a hyperpolarized membrane (Fig. 4B).

#### Myc-overexpressing clones

When Myc is overexpressed in random clones within wing discs, the clones outcompete the neighboring wild-type cells and expand within the tissue (Moreno and Basler, 2004). Although Myc-overexpressing clones represent a well-established model of cell competition rather than tumors per se, understanding how precancerous cells over-proliferate at the expense of normal cells might help identify crucial requirements for the early phase of tumor formation.

Regarding the roles in cell metabolism, elevated Myc upregulates glycolytic genes including LDH (de la Cova et al., 2014) in a cell-autonomous manner, promotes mitochondrial biogenesis but not fusion (de la Cova et al., 2014; Li et al., 2005) and causes an overall increase in ΔΨ_m_ (Banreti and Meier, 2020). When overexpressed in half of the wing disc rather than limited to clones, Myc leads to massive production of ROS (Sollazzo et al., 2018) – although ROS levels in Myc-overexpressing clones have not been reported. Notably, Myc-overexpressing clones trigger a non-autonomous metabolic change in the surrounding wild-type cells (Banreti and Meier, 2020). In the clones, Myc upregulates the Nmdar2 subunit (hereafter referred to as NR2) of the *N*-methyl-D-aspartate (NMDA) receptor, creating an imbalance of NR2 levels between the Myc-overexpressing and the adjacent wild-type cells (Banreti and Meier, 2020). This disparity triggers a JNK/PDK/PDH/JNK-dependent positive feedback loop in the wild-type cells, diverting pyruvate from the TCA cycle towards the production and secretion of lactate that is then taken up by Myc-overexpressing clones (Banreti and Meier, 2020).

Why do Myc-overexpressing cells take up so much lactate? Intracellular lactate may be oxidized to pyruvate in mitochondria to serve as energy source or used as a gluconeogenic substrate (de la Cruz-López et al., 2019). Recent findings suggest that melanoma cells, especially the ones with high metastatic potential, favor lactate import to stimulate the pentose phosphate pathway and counteract oxidative stress (Tasdogan et al., 2020). If Myc-overexpressing clones also display elevated ROS, would lactate import be a protective mechanism to ensure cell survival in the cell competition setting? Although these questions – yet – remain unanswered, Myc-overexpressing clones might represent a novel model to study cell-to-cell lactate shuttle.

#### Hipk neoplasia

Unlike most epithelial neoplastic tumor models that display high levels of ROS, Hipk neoplasia has a distinct mitochondrial profile – the accumulation of hyperfused and hyperpolarized mitochondria as well as a low production of ROS (Wong et al., 2020a). These mitochondrial changes are Myc dependent. Given that Myc itself is insufficient to cause mitochondrial fusion (de la Cova et al., 2014), additional not-yet-identified mechanisms might drive the mitochondrial changes in Hipk tumors. One potential target is Yki, which is positively regulated by Hipk (Chen and Verheyen, 2012; Poon et al., 2012). Activated Yki causes hyperplasia through promoting mitochondrial hyperfusion (Nagaraj et al., 2012). Thus, it is conceivable that Myc, Yki and other oncogenes contribute to the changes in mitochondrial dynamics, abundance and energetics within Hipk tumors.

Surprisingly, mitochondrial hyperfusion is functionally irrelevant to Hipk tumor growth or membrane hyperpolarization (Wong et al., 2020a), a fact that is contrary to what is observed in the Yki hyperplasia model (Nagaraj et al., 2012) and in *brat-RNAi* brain tumors (Bonnay et al., 2020) described below. This highlights the fact that mitochondrial dynamics and energetics can be controlled separately. Such uncoupling is also observed in fly neurons, where neuronal health depends on functional mitochondria – regardless of whether the mitochondria are fused or fragmented (Trevisan et al., 2018). Another point illustrated by the Hipk tumor model is that the tumorigenic outcomes vary when different ETC subunits are inhibited (Wong et al., 2020a). Whereas knockdown of *Pdsw* (encoding an ETC complex I subunit) produces mild ROS and abrogates Hipk tumor growth, knockdown of *ATPsynβ* (encoding an ETC complex V subunit) produces high levels of ROS, and potentiates JNK activation and tumor invasion. Variation in knockdown efficiencies aside, this might reflect a unique susceptibility of each ETC complex subunit to electron leaks.

#### Neural stem cell tumors

Not only can carcinomas be modeled in *Drosophila*, tumors originated from fly stem cells, such as neuroblasts (neural stem cells, NSCs) and intestinal stem cells (ISCs), can also be replicated. Normally, stem cells divide asymmetrically to both self-renew and differentiate into specialized cell types. Emerging evidence indicates that cancer stem cells exist within tumors, which might account for recurrence and metastasis despite initial chemo- or radiotherapy (Yang et al., 2020). Thus, understanding the characteristics of stem cell tumors is of great significance in the context of cancer treatment efficacy.

Larval brain stem cell tumors, which no longer differentiate but continue to proliferate and retain stem cell-like properties, can be induced by loss of either the RNA-binding protein brain tumor (Brat) or the transcription factor prospero (Pros) (Betschinger et al., 2006). Recently, three independent studies explored the metabolic requirements of fly brain tumorigenesis (Bonnay et al., 2020; Genovese et al., 2019; Van Den Ameele and Brand, 2019). In particular, the mitochondrial profile of *brat-RNAi* brain tumors was characterized in detail. *brat-RNAi* brain tumors feature mitochondrial hyperfusion, membrane hyperpolarization, increased oxygen consumption, robust NAD^+^ regeneration and utilization of glutamine in the TCA cycle, suggesting elevated OxPhos (Bonnay et al., 2020). Single-cell RNA sequencing has identified heterogeneous populations of *brat-RNAi* brain tumor cells, with a small population featuring high levels of proliferation and OxPhos, whereas the majority exhibiting a less-proliferative signature accompanied by elevated aerobic glycolysis and a high extent of oxidative stress (Bonnay et al., 2020). Failure in NAD^+^ regeneration upon inhibition of mitochondrial fusion or TCA/OxPhos leads to a reduction in tumor size (Bonnay et al., 2020; Genovese et al., 2019; Lee et al., 2013; Van Den Ameele and Brand, 2019), suggesting that tumor growth is primarily supported by the small population of tumor cells with an active mitochondrial metabolism.

When transplanted into the abdomen of an adult fly host, larval brain tumors can continue to grow and metastasize (Beaucher et al., 2007a). After transplantation, *brat-RNAi* brain tumors induce more ROS than their non-transplanted counterparts (Hussain et al., 2017). Inhibition of mitochondrial respiration no longer suppresses the growth of the transplanted tumor. Rather, the transplanted tumors harness enzymes involved in glyceroneogenesis and/or gluconeogenesis to regenerate NAD^+^ and support growth. Thus, both studies show that brain tumors display a changing metabolic dynamics over time and use various mechanisms to maintain redox balance.

#### Intestinal stem cell tumors

Similar to NSCs, ISCs asymmetrically divide to self-renew and give rise to differentiated cells including enterocytes and endocrine cells (Jiang and Edgar, 2012). ISC tumors induced by activated Ras signaling show little overproduction of ROS and increased mitochondrial abundance when compared with normal ISCs (Ma et al., 2016; Morris et al., 2020). Also, ND-75 is required for Ras-activated ISC over-proliferation (Ma et al., 2016), illustrating the importance of functional mitochondria. Caliban (Clbn) is a tumor suppressor that regulates mitochondrial dynamics and energetics (Dai et al., 2020). Loss of Clbn not only results in mitochondrial fission, membrane depolarization and high levels of ROS but also potentiates the invasiveness of *Ras^V12^*-activated ISC tumors. Thus, similar to the epithelial tumor models discussed above, both functional and dysfunctional mitochondria – paradoxically – seem to support tumor progression. In another ISC tumor model induced by *Notch-RNAi*, loss of mitochondrial pyruvate carrier (MPC) to inhibit oxidative pyruvate metabolism further increases proliferation (Schell et al., 2017), suggesting that mitochondrial metabolism, when perturbed, is flexible and continues to fuel growth, possibly by triggering aerobic glycolysis and/or switching to metabolize other substrates like glutamine. *Notch-RNAi* ISC tumors display a gradual increase in ROS over time. Moreover, when coupled with loss of β-integrin, *Notch-RNAi* ISC tumors become metastatic and produce high levels of ROS (Chen et al., 2021), reflecting distinct metabolic states as the tumor progresses.

Finally, another key aspect of mitochondrial metabolism – mitochondrial quality control, i.e. mitophagy, mediated by phosphatase and tensin homolog (PTEN)-induced kinase 1 (PINK1) and/or Parkin – has been linked to proliferation of both NSC and ISC (Koehler et al., 2017; Lee et al., 2013). Therefore, it would be interesting to see whether PINK1 and/or Parkin participate in epithelial tumor progression.

## Conclusion

In this Review, we have highlighted the recent *Drosophila* studies that contributed to the thriving field of cancer metabolism, which can be summarized in several take-home messages. First, fly tumors exhibit a dynamic range of metabolic profiles as they progress. Second, to rewire metabolism, fly tumors use various distinct strategies that involve feedback circuits, nutrient-sensing machinery and non-autonomous regulation. Third, the metabolic profiles of fly tumors, especially their mitochondrial metabolism, are far more heterogeneous than previously thought. Looking forward, we are optimistic that the metabolic vulnerabilities, inter-cellular and inter-organ communications identified in fly tumor models are of great significance regarding anti-cancer drug development and/or cancer diagnosis. Indeed, in many cases, we have already seen parallels between tumor models in fly and others organisms, including mouse xenografts and patient-derived xenografts, validating the use of *Drosophila* to tackle the genetic and metabolic complexity of human cancers. With the emerging single-cell omics and new non-invasive metabolic probes – genetically and through imaging, respectively – future studies are poised to discern tumor cell populations with specific metabolic requirements, such that targeted therapeutic efficacy can be achieved with minimal adverse effects.
